# Ultrasound-guided acupotomy for cervical spondylosis: a systematic review and meta-analysis based on GRADE quality assessment

**DOI:** 10.3389/fpain.2025.1654265

**Published:** 2025-11-14

**Authors:** Zhang Lijian, Zhang Zhen, Yuan Yuan, Zhang Nachuan

**Affiliations:** 1 Orthopedics Department, Changzhi People’s Hospital, Changzhi, China; 2 Department of Physical Education, Changzhi University, Changzhi, China; 3 School of Acupuncture and Tuina, Nanjing University of Chinese Medicine, Nanjing, China

**Keywords:** cervical spondylosis, ultrasound-guided acupotomy, meta-analysis, systematic review, grade

## Abstract

**Objective:**

This study aimed to evaluate the effectiveness of ultrasound-guided acupotomy (UgA) in treating Cervical spondylosis (CS), particularly in pain relief, improvement in cervical range of motion (CROM), and overall clinical efficacy, through a systematic review and meta-analysis based on GRADE quality assessment.

**Methods:**

Following PRISMA guidelines, we searched databases including PubMed, Embase, Cochrane Library, Web of Science, and CNKI, Wanfang, Weipu, and Sinomed, identifying 33 randomized controlled trials (RCTs). Inclusion criteria were: patients aged 18–70 with a diagnosis of CS, intervention with UgA, and control groups receiving placebo, physical therapy, or other conventional treatments. Primary outcomes included clinical effective rate and Visual Analog Scale (VAS) for pain, while secondary outcomes encompassed Neck Disability Index (NDI), CROM, and mean flow velocity of vertebral and basilar arteries (MFV-VA/BA). Study quality was assessed using the Cochrane Risk of Bias 2.0 tool, and meta-analysis was conducted using Stata 15.0. The GRADE approach was used to evaluate evidence quality.

**Results:**

Meta-analysis revealed that UgA significantly improved the clinical effective rate compared to control treatments (RR = 1.17, 95% CI: 1.13–1.21), with low heterogeneity (*I*^2^ = 12%). UgA also demonstrated significant pain reduction (WMD = −0.96, 95% CI: −1.25 to −0.67), albeit with high heterogeneity (*I*^2^ = 91.6%). For secondary outcomes such as NDI, CROM, and MFV-VA/BA, UgA showed moderate improvements, but with considerable heterogeneity. GRADE assessment indicated high-quality evidence for the clinical effective rate, while evidence for VAS, NDI, and CROM was rated as low or very low due to heterogeneity and publication bias.

**Conclusion:**

UgA shows superior efficacy for pain and disability in cervical spondylosis compared to non-UgA and other acupuncture related therapies. However, heterogeneity and potential publication bias exist. It requires skilled practitioners and real-time ultrasound guidance for treatment. Future multinational randomized trials with standardized protocols are needed.

**Systematic Review Registration:**

https://www.crd.york.ac.uk/PROSPERO/PROSPERO, PROSPERO CRD42025649835.

## Introduction

Cervical spondylosis (CS), or cervical degenerative disc disease, is a prevalent musculoskeletal disorder of the cervical spine1 (1, 2), driven by aging and modern lifestyle factors such as prolonged use of smartphones, computers, and other electronic devices (3). The global prevalence of CS estimated that 30% to 50% of individuals over the age of 40 experience some form of cervical degeneration (4, 5). Furthermore, the disease is considered a significant cause of disability, affecting up to 20% of individuals in their lifetime (5).

CS is characterized by neck pain and stiffness, often accompanied by radicular pain, numbness, tingling, headache, nausea, gastrointestinal discomfort, blurred vision, tinnitus, hypomnesia, palpitations, and, in severe cases, motor and sensory deficits in the upper limbs (2, 6). With aging, degenerative changes develop, including intervertebral disc herniation, osteophyte formation, and ligament calcification. These changes can cause nerve root compression, spinal cord impingement, and inflammation (7). This degeneration results in reduced flexibility of the cervical spine, leading to mechanical stress and causing pain, dysfunction, and neurological deficits (8). Factors such as poor posture, repetitive neck movements, and trauma may accelerate the degenerative process, exacerbating the symptoms of CS (9).

CS can be classified into different clinical subtypes based on the nature and severity of the symptoms. These include cervical axial syndrome, cervical radiculopathy, cervical myelopathy, and cervicogenic headache (10). The clinical presentation can vary significantly depending on the type and location of the degenerative changes. Diagnosis is typically made based on a comprehensive assessment that includes a thorough clinical history, physical examination, and imaging studies, such as x-rays, magnetic resonance imaging (MRI), or computed tomography (CT) scans (11). Treatment options for CS include conservative measures such as physical therapy, pharmacological management (analgesics, anti-inflammatory drugs, muscle relaxants), and interventional procedures like epidural steroid injections (12). If conservative treatments fail, surgery may be considered. Options include discectomy and spinal fusion.

Given the limitations of conventional treatments, there has been growing interest in exploring alternative and complementary therapies for CS. One such treatment is acupuncture, which has been used in Traditional Chinese Medicine (TCM) for thousands of years to alleviate pain and promote healing. In recent years, acupuncture techniques have advanced. A modern approach, known as “acupotomy therapy,” has been introduced (13, 14). The procedure involves inserting an acupotomy at the site of pain or spasm to release soft-tissue tension. This improves blood circulation and reduces inflammation (15). Ultrasound-guided Acupotomy (UgA) has shown promise in treating CS and its associated symptoms, such as pain, muscle tension, and reduced mobility (16). The ultrasound guidance ensures accurate localization of the anatomical structures and facilitates the targeting of specific regions of the cervical spine affected by degenerative changes. UgA delivers mechanical stimulation that may restore tissue function by promoting collagen synthesis and activating fibroblasts (17).

Compared with landmark-based (non-guided) acupotomy or other conservative interventions, ultrasound guidance provides real-time visualization of the target soft tissues and adjacent neurovascular structures, enabling more precise release and safer manipulation (18). This precision reduces repeated passes and iatrogenic injury while improving the likelihood of complete adhesiolysis at the affected cervical levels. Despite the promising results of UgA, there remains a lack of consensus regarding its efficacy and safety compared to other conventional treatments. The purpose of this study is to conduct a systematic review and meta-analysis to evaluate the effectiveness of UgA in treating CS, specifically focusing on pain relief, improvement in range of motion, and overall treatment efficacy.

## Methods

### Study design and protocol

This study adheres to the guidelines established by the Preferred Reporting Items for Systematic Reviews and Meta-Analyses (PRISMA) statement (19). A systematic review and meta-analysis were conducted to evaluate the effectiveness of UgA therapy in treating CS, focusing on pain relief, improvement in the range of motion, and overall clinical efficacy. The protocol for this systematic review and meta-analysis was registered with PROSPERO (CRD42025649835) prior to the commencement of the study.

### Literature search

A comprehensive literature search was performed in the following databases: PubMed, Embase, Cochrane Library, Web of Science, CNKI, Weipu, Sinomed and Wanfang. We searched Mesh terms related to “Ultrasonography” and “Spondylosis”. Then we applied a subject-heading + free-text + near-synonym/variant strategy across title, abstract, and keyword fields, such as “ultrasound”, “ultrasonic imaging”, “ultrasonographic imaging”, “ultrasonics”, “cervical spondylosis”, “cervical syndrome”, “cervical spondylopathy”, “acupotomy”, “acupotomology”, “needle knife”, and “needle-knife”. The search was conducted without language restrictions. The literature search was updated through October 2024. The search strategy for PubMed can be found in Supplementary Material.

### Eligibility criteria

Inclusion Criteria:

1. Study Design: Only randomized controlled trials (RCTs) were included, as RCTs minimize selection bias and provide the highest level of evidence for evaluating intervention efficacy.
2. Population: Studies must involve human participants aged 18–70 years with a clear diagnosis of CS (20), regardless of the subtype or severity of the disease. Studies that include participants with CS as the primary condition were eligible for inclusion.
3. Intervention: The intervention must be Ultrasound-guided Acupotomy (UgA) for the treatment of CS. The studies must compare this intervention with a placebo, standard physical therapy, pharmacological treatments, or other conventional therapeutic modalities.
4. Primary Outcomes: Studies must report at least one of the following primary outcomes:

Efficacy: Improvement in clinical efficacy, as defined by the number of patients achieving “cure,” “significant improvement,” or “effective response.”

Pain Reduction: Changes in Visual Analog Scale (VAS) scores for pain relief.

1. Secondary Outcomes: Studies must report at least one of the following secondary outcomes:

Neck Disability Index (NDI): Evaluation of functional disability related to CS.

Cervical Range of Motion (CROM): Improvement in the cervical spine's range of motion.

Symptom and Function Assessment (SFA): Assessments that evaluate changes in symptoms and functional status.

Mean Flow Velocity of Vertebral and Basilar Arteries (MFV-VA/BA): Measurement of the average flow velocity in the vertebral and basilar arteries as assessed by Doppler ultrasound or similar techniques.

1. Publication Type: Studies must be published in peer-reviewed journals with available full-text data. There were no restrictions based on language.

Exclusion Criteria:

1. Study Design: Studies that are not randomized controlled trials (RCTs) (e.g., observational studies, case series, case reports) were excluded.
2. Population: Studies that involve participants outside the age range of 18–70 years or those with conditions other than CS were excluded. Studies focusing on subgroups with additional severe comorbidities or disorders unrelated to CS were also excluded.
3. Intervention: Studies that do not use UgA therapy as an intervention or studies that do not include a valid control group (e.g., placebo, conventional treatments) were excluded.
4. Outcomes: Studies that do not report on the primary outcomes of efficacy or VAS scores, or fail to report any of the predefined secondary outcomes (NDI, CROM, CASCS, or MFV-VA/BA) were excluded.
5. Data Quality: Studies that do not provide sufficient data for statistical analysis (e.g., incomplete outcome reporting, lack of baseline or follow-up data) or those with significant methodological flaws that cannot be addressed were excluded.
6. Publication Type: Conference abstracts, editorials, letters to the editor, and case reports were excluded due to insufficient data for inclusion in a meta-analysis.

### Study selection and data extraction

The study selection and data extraction processes were carried out by two independent reviewers (LJ and Z). In the first stage, they screened titles and abstracts, followed by a full-text review in the second stage. Any discrepancies between the reviewers were resolved through discussion, and if consensus could not be reached, a third reviewer (YY) was consulted. Only studies that met the predefined inclusion criteria were included in the final analysis. A flowchart depicting the study selection process was created according to the PRISMA guidelines.

After selecting the studies, the two reviewers independently extracted relevant data using a standardized form. The extracted data included study characteristics such as the first author, year of publication, study design, sample size, and follow-up duration. Patient characteristics, including age, sex, disease duration, and baseline severity of CS, were also collected. Details of the interventions, such as the type of acupotomy therapy, including needle specifications, ultrasound guidance, frequency, and duration of treatment, along with the comparison group (placebo, pharmacological treatment, or physical therapy), were documented. The primary and secondary outcomes were recorded, including pain reduction (measured by VAS), improvement in range of motion, and overall clinical efficacy as reported in terms of cure, significant improvement, or effective response. Lastly, any adverse events related to UgA therapy, such as hematomas or infections, were extracted for analysis.

### Risk of bias assessment

The risk of bias in the included studies was assessed using the Risk of Bias 2.0 (ROB 2.0) tool for randomized controlled trials (RCTs) (21). This tool evaluates bias in five key domains: randomization process, deviations from intended interventions, missing outcome data, measurement of the outcome, and selection of the reported results. Each study was assessed for risk of bias in each domain, and classified as having low, high, or some concerns regarding bias. Discrepancies between reviewers were resolved through discussion and consensus.

## Statistical analysis

Meta-analysis was conducted using Stata version 15.0. For continuous outcomes (e.g., VAS scores and NDI), the mean difference (MD) with 95% confidence intervals (CI) was calculated. For other continuous outcomes, such as cervical range of motion, standardized mean differences (SMD) were used. For dichotomous outcomes (e.g., clinical efficacy), the risk ratio (RR) with 95% CI was used. All analyses were conducted using a random-effects model. Heterogeneity among studies was assessed using the *I*^2^ statistic, with *I*^2^ values of 25%, 50%, and 75% indicating low, moderate, and high heterogeneity, respectively. A fixed-effect model was used for data synthesis when heterogeneity was below 50%; otherwise, a random-effects model was applied. Subgroup analyses were performed to explore potential sources of heterogeneity, including study design, type of comparison group, and follow-up duration. Publication bias was evaluated using funnel plots and Egger's test for studies with more than 10 included trials. Sensitivity analysis was conducted by excluding studies with a high risk of bias to assess the robustness of the results.

### Certainty of evidence

The Grading of Recommendations, Assessment, Development, and Evaluations (GRADE) approach was used to assess the certainty of evidence for the primary outcomes (22). The quality of evidence was rated as high, moderate, low, or very low, based on factors such as study limitations, inconsistency, indirectness, imprecision, and publication bias.

## Results

### Included studies and basic characteristics

**Figure 1:**
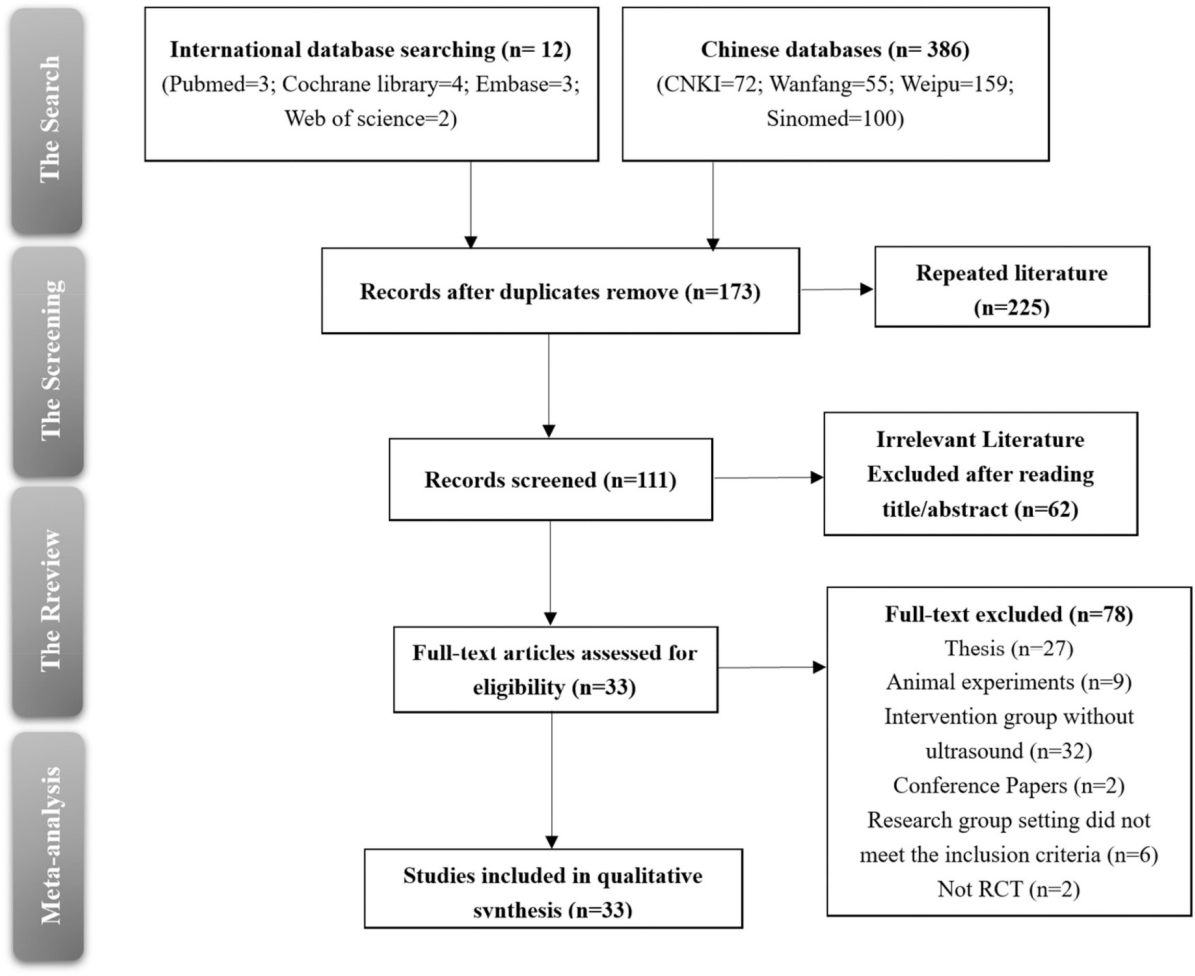
Literature screening and inclusion process.

In this systematic review, a total of 33 randomized controlled trials (RCTs) were included after a thorough screening and selection process. The search initially yielded 12 records from international databases (PubMed, Cochrane Library, Embase, and Web of Science) and 386 records from Chinese databases (CNKI, Wanfang, Weipu, and Sinomed). After removing duplicates, 173 records were retained for further screening. Of these, 111 records were reviewed, and 33 studies met the inclusion criteria for qualitative synthesis (Figure 1).

The included studies focused on the effect of UgA interventions for CS compared to various control treatments. The characteristics of the participants in these studies ranged from 20 to 100 individuals per trial, with a mean age ranging from 34 to 79 years. The majority of participants were middle-aged and older adult individuals, reflecting the typical demographic affected by CS. The specific characteristics of the study are shown in Table 1.

**Table 1:** Characteristics of included studies.

Study	Characteristics of patients	Intervention group	Control group	Outcome
Pu 2023 (23)	80/80; 55.6 ± 10.5/52.5 ± 11.5(y)	UgA. Blocking solution: 1.0 mL of Compound Betamethasone Injection (5 + 2 mg), 1.5 mL of 2% lidocaine hydrochloride (30 mg), and 3.5 mL of 0.9% sodium chloride, totaling 6 mL for standby. Acupotomy: Hanqing brand disposable sterile injection acupotomy was used, with a specification of 0.9*85 mm. 2 weeks.	UgNRB. Blocking solution: 1.0 mL of Compound Betamethasone Injection (5 + 2 mg), 1.5 mL of 2% lidocaine hydrochloride (30 mg), and 3.5 mL of 0.9% sodium chloride, totaling 6 mL for standby. 2 weeks.	Effective rate; VAS; NDI.
Cai 2019 (24)	40/40; 49.3/48.9(y)	UgA. Ultrasound guidance; acupotomy: No. 3 Type I Needle Knife. 3 weeks.	UgNRB. Ultrasound guidance; using a 0.7 × 80 mm common puncture needle, 2 mL of nerve block solution (formula: 2% lidocaine 3 mL + dexamethasone 5 mg + methylcobalamin 0.5 mg + physiological saline 5 mL) was injected. 3 weeks.	Effective rate; VAS; CROM.
Deng 2016 (25)	30/30; 52 ± 16/51 ± 18(y)	UgA + UgNRB. Ultrasound equipment: portable ultrasound machine from sonosite (model: MicroMAXX), high-frequency ultrasound probe (6–13 MHz); needle knife: Hanzhang No. 4 needle knife; anti-inflammatory and analgesic solution preparation: 2% lidocaine 1 mL, methylcobalamin 1 mL, compound betamethasone 7 mg, a total of 3 mL. 4–6 weeks.	Non-UgA + NRB. No ultrasound guidance. Needle knife: Hanzhang No. 4 needle knife; anti-inflammatory and analgesic solution preparation: 2% lidocaine 1 mL, methylcobalamin 1 mL, compound betamethasone 7 mg, a total of 3 mL. 4–6 weeks.	Effective rate; VAS.
Ding 2022 (26)	41/41; 67.82 ± 3.90/66.95 ± 3.47(y)	UgA + PRF. A No. 4 needle knife was used for acupotomy treatment. For cervical nerve root pulsed radiofrequency, the parameters were set as follows: frequency 2 Hz, pulse width 20 ms, temperature 42°C, and time 15 min. 8 weeks.	Non-UgA. No ultrasound guidance. No. 4 needle Knife. 8 weeks.	Effective rate; VAS; CROM; MFV-VA/BA.
Du 2023 (27)	37/32; 46.4 ± 10.2/48.9 ± 9.8(y)	UgA + UgNRB. Ultrasound equipment: color Doppler ultrasound diagnostic instrument (WISONIC Navi model, 9−12 MHz); needle knife: 0.6 × 50 Hanzhang needle knife; liquid preparation: 2% lidocaine 2 mL + compound betamethasone injection 0.3 mL + adenosylcobalamin 1.5 mg + 0.9% sodium chloride injection 6 mL to prepare a mixed liquid of 8 mL, injection volume 2 mL. 2 weeks.	Non-UgA + NRB. No ultrasound guidance. Needle knife: 0.6 × 50 Hanzhang needle knife; liquid preparation: 2% lidocaine 2 mL + compound betamethasone injection 0.3 mL + adenosylcobalamin 1.5 mg + 0.9% sodium chloride injection 6 mL to prepare a mixed liquid of 8 mL, injection volume 2 mL. 2 weeks.	Effective rate; VAS; NDI.
Fu 2024 (28)	30/30; 50.31 ± 8.03/50.05 ± 8.12(y)	UgA + moxibustion. 1% lidocaine was administered for local anesthesia. Under the guidance of a portable ultrasound device, a No. 4 needle knife was vertically inserted. Thermal moxibustion. 2 weeks.	Non-UgA + moxibustion. 1% lidocaine was used for local anesthesia. A No. 4 needle knife was then vertically inserted at the superior and inferior borders of the vertebral spinous process. Thermal moxibustion. 2 weeks.	Effective rate.
Gao 2021 (29)	60/60; 46.08 ± 11.55/47.35 ± 10.81(y)	UgA. Treatment was performed using a LeJiu brand needle knife (0.5 mm × 50 mm) under the guidance of a portable color ultrasound device (6–13 MHz). 15 days.	Non-UgA. Using a LeJiu brand needle knife (0.5 mm × 50 mm) under the guidance of a portable color ultrasound device. 15 days.	Effective rate; SFA.
Hu 2021 (30)	44/44; 55.09 ± 2.67/55.14 ± 2.83(y)	UgA. Under ultrasound guidance, anesthesia was performed with 1% lidocaine; a No. 4 needle knife was used to insert the needle. NA.	Non-UgA. Anesthesia was performed with 1% lidocaine; a No. 4 needle knife was used to insert the needle. NA.	Effective rate.
Huang 2023 (31)	40/40; 55.25 ± 7.86/54.76 ± 7.69(y)	UgA. Under ultrasound guidance (Sonosite M-Turbo 4th Generation Portable Ultrasound, 6–13 MHz), a sterile needle knife (size 0.6 mm × 40 mm) was used to insert the needle. 4 weeks.	Non-UgA. A sterile, Hanzhang brand needle knife (0.6 mm × 40 mm) was used for needle insertion. 4 weeks.	Effective rate; VAS; NDI; CROM.
Huang 2023 (1) (32)	20/20; 43.40 ± 11.09/40.20 ± 11.83(y)	UgA. Under ultrasound guidance, a disposable No. 4 Xijiu brand needle knife was used for the operation. 15 days.	UgA (T shape). A disposable Xijiu brand No. 4 needle knife was used for the procedure. 15 days.	Effective rate; VAS; CROM.
Jian 2020 (33)	23/23; 48.5 ± 2.3/48.0 ± 2.2(y)	UgA. The needle knife procedure was performed under ultrasound guidance. 4 weeks.	Non-UgA. Blind needle knife release treatment. 4 weeks.	VAS; SFA.
Jiang 2020 (34)	32/32; 34.26 ± 4.31/34.21 ± 4.36(y)	UgA. Under ultrasound guidance, a No. 4 needle knife was used to perform the procedure. 4 weeks.	ACU. 0.30 mm × 25.00 mm acupuncture needle. 4 weeks.	Effective rate; VAS; NDI; SFA.
Jiang 2021 (35)	50/50; 34.89 ± 8.56/35.71 ± 8.27(y)	UgA. Ultrasound guidance (Philips iu22 model, 5–11 MHz) was used during the procedure with a No. 4 disposable sterile Type I needle knife. 2 weeks.	Non-UgA. The procedure was performed using a No. 4 disposable sterile Type I needle knife without ultrasound guidance and under non-visual conditions. 2 weeks.	Effective rate; VAS; NDI; SFA.
Li 2022 (36)	53/53; 79.90 ± 11.66/50.13 ± 12.38(y)	UgA + ACU. Using a No. 4 needle knife under ultrasound guidance (DE-PF542, 7–12 MHz), the procedure was performed following local anesthesia with 1% lidocaine. Disposable sterile acupuncture needles Specifications 0.3 × 40 mm. 3 weeks.	ACU. Disposable sterile acupuncture needles Specifications 0.3 × 40 mm. 3 weeks.	Effective rate; VAS.
Li 2015 (37)	30/30; 52 ± 16/51 ± 18(y)	UgA + UgNRB. A No. 4 needle knife was used for the procedure under real-time ultrasound guidance (MicroMAXX portable ultrasound machine from Sonosite, 6–13 MHz), and 0.5–1 mL of anti-inflammatory and analgesic medication was injected. 8 weeks.	UgNRB. MicroMAXX portable ultrasound machine from Sonosite, 6–13 MHz. Anti-inflammatory analgesic 0.5–1 mL. 8 weeks.	Effective rate; VAS.
Li 2020 (38)	28/28; NA	UgA + TCM. 1.0% lidocaine was used for local infiltration anesthesia at each point, followed by a procedure using a No. 4 needle knife under ultrasound guidance (Mindray M7 Color Doppler Ultrasound Diagnostic Instrument, 9–12 MHz). Jia Wei Ge Gen Decoction. 18 days.	ACU. Huatuo brand 0.3 mm*40 mm (1.5 inches) disposable acupuncture needle. 18 days.	Effective rate.
Liu 2024 (39)	30/30; 53.33 ± 2.25/49.73 ± 2.20(y)	UgA. Ultrasonic equipment: high-frequency linear array probe, 7.51–12 MHz. A 2 mL solution of 2% lidocaine and 8 mL of normal saline was used as the anesthetic, and a disposable Xijiu brand Type I No. 4 needle knife was selected for the procedure. 3 weeks.	ACU. Acupuncture needles. 3 weeks.	VAS; NDI.
Liu 2018 (40)	50/50; 43.53 ± 10.59/41.22 ± 10.39(y)	UgA. Ultrasound-guided needle knife release procedure. 2 weeks.	Non-UgA. The needle knife release procedure was performed without ultrasound guidance. 2 weeks.	Effective rate; VAS; CROM.
Liu 2021 (41)	100/100; 37.30 ± 7.95/35.19 ± 9.61(y)	UgA. The procedure was performed with a Huaxia brand No. 4 Type I needle knife under ultrasound guidance (DIMENSIONAL Antares, LOGIQ E9, 9–14 mHz). 2 weeks.	Non-UgA. The procedure was performed using a Huaxia brand No. 4 Type I needle knife without ultrasound guidance and under non-visual conditions. 2 weeks.	VAS; SFA.
Luo 2023 (42)	87/87; 70.06 ± 1.12/69.58 ± 1.07(y)	UgA + PRF. Under ultrasound guidance, a No. 4 needle knife was used for release treatment; subsequently, a Beijing R-2000B radiofrequency treatment device was used, with stimulation set at 0.5 V and 1.0 V respectively, to perform sensory and motor tests, inducing sensory and motor responses in the corresponding nerve innervation area. Following this, the pulsed radiofrequency frequency and pulse width were adjusted to 2 Hz and 20 ms respectively, the temperature was set to 42°C, and radiofrequency treatment was administered for 15 min. 3 weeks.	Non-UgA. Without ultrasound guidance, a No. 4 needle knife was used to perform the release procedure. 3 weeks.	Effective rate; VAS; NDI; CROM; MFV-VA/BA.
Qi 2018 (43)	41/41; 67.85 ± 5.64/66.85 ± 5.23(y)	UgA. Under ultrasound guidance, 1% lidocaine was used for anesthesia, a No. 4 needle knife was inserted, and after its removal, anti-inflammatory and analgesic medication was injected at the wound site. NA.	Non-UgA. Without ultrasound guidance, 1% lidocaine was used for anesthesia, a No. 4 needle knife was inserted, and after its removal, anti-inflammatory and analgesic medication was injected at the wound site. NA.	VAS.
Quan 2024 (44)	46/46; 47.23 ± 5.11/46.59 ± 4.37(y)	UgA. Under ultrasound guidance, 6 mL of a 1% lidocaine solution (prepared by diluting 2% lidocaine 1:1 with saline) was administered for local anesthesia, followed by needle knife release. 2 weeks.	Non-UgA. Without ultrasound guidance, 6 mL of a 1% lidocaine solution (prepared by diluting 2% lidocaine 1:1 with saline) was administered for local anesthesia, followed by needle knife release. 2 weeks.	VAS; NDI; CROM.
Wang 2014 (45)	30/30; 42 ± 10/43 ± 12(y)	UgA. Under portable ultrasound guidance (Ultrasound equipment: Sonosite MicoroMaxx portable ultrasound machine and high-frequency imaging probe, 6–13 mHz), a No. 4 Hanzhang needle knife was used for release, followed by an injection of anti-inflammatory and analgesic solution (1% lidocaine 1 mL and compound betamethasone 3 mg) at the original needle insertion site. 8 weeks.	Non-UgA. Without ultrasound guidance, a No. 4 Hanzhang needle knife was used for release, followed by an injection of anti-inflammatory and analgesic solution (1% lidocaine 1 mL and compound betamethasone 3 mg) at the original needle insertion site. 8 weeks.	Effective rate; VAS.
Wang 2020 (46)	50/50; 46.77 ± 6.32/45.98 ± 7.41(y)	UgA + PRF. Under ultrasound guidance (PHILIPS, HD15, 3–12 MHz), a Huaxia brand No. 4 Type I needle knife was used for treatment, followed by radiofrequency therapy (42°C, 2 Hz, 20 ms, 120 s). 2 weeks.	Non-UgA. Without ultrasound guidance, treatment was performed using a Huaxia brand No. 4 Type I mini-needle knife. 2 weeks.	Effective rate; VAS; CROM.
Wang 2020 (1) (47)	28/28; 50.54 ± 7.96/48.91 ± 12.69(y)	UgA. Under ultrasound guidance (10–15 Hz), after local infiltration anesthesia with 1% lidocaine (prepared by diluting 2% lidocaine at a 1:1 ratio), a Huaxia brand No. 4 Type I needle knife was used for treatment. NA.	Non-UgA. Without ultrasound guidance, local infiltration anesthesia was performed using 1% lidocaine (prepared by diluting 2% lidocaine at a 1:1 ratio), followed by treatment with a Huaxia brand No. 4 Type I needle knife. NA.	Effective rate; VAS; NDI.
Wang 2017 (48)	40/40; 51.2 ± 6.5/49.5 ± 11.5(y)	UgA. 1% lidocaine was used for local infiltration anesthesia at the insertion point; under ultrasound guidance (PHILIPS, CX50, 6–12 MHZ), a No. 4 needle knife was used for the procedure, and after needle removal, 1 mL of anti-inflammatory and analgesic solution was injected. NA.	Non-UgA. 1% lidocaine was used for local infiltration anesthesia at the insertion point; without ultrasound guidance, a No. 4 needle knife was used for the procedure, and after needle removal, 1 mL of anti-inflammatory and analgesic solution was injected. NA.	Effective rate; VAS; MFV-VA/BA.
Yuan 2012 (49)	30/30; 52 ± 7.7/50 ± 9.5(y)	UgA. The procedure was performed using a GE LOGIC 7 color Doppler ultrasound system with a 7.5–12 MHz transducer. 6 weeks.	Non-UgA. Palpation and localization were performed using anatomical landmarks. 6 weeks.	Effective rate.
Yuan 2021 (50)	30/30; 55.85 ± 1.42/54.75 ± 1.16(y)	UgA + UgNRB + TCM. Ultrasound equipment: Siemens (model: ACUSON P500) portable color Doppler ultrasound instrument (probe rate: 5–10 MHz); anti-inflammatory and analgesic solution: 2% lidocaine 5 mL + dexamethasone 5 mg + methylcobalamin 0.5 mg + normal saline, diluted to 10 mL, 2 mL injected into each intervertebral foramen; needle knife: Hanzhang brand type I No. 4 needle knife; TCM: Jingfukang granules. 4 weeks.	Non-UgA + NRB + TCM. Anti-inflammatory and analgesic solution: 2% lidocaine 5 mL + dexamethasone 5 mg + methylcobalamin 0.5 mg + normal saline, diluted to 10 mL, 2 mL injected into each intervertebral foramen; TCM: Jingfukang granules. 4 weeks.	Effective rate.
Zhang 2020 (51)	30/30; 62.40 ± 3.05/54.34 ± 4.25(y)	UgA + ACU. Ultrasound equipment: S40 color Doppler ultrasound diagnostic instrument (probe frequency: 7–12 MHz); anesthetic: 1% lidocaine; acupotomy: Huayou brand No. 4 acupotomy (1.0 mm × 50 mm); acupuncture: Huatuo brand 0.3 mm × 40 mm disposable sterile acupuncture needle. 3 weeks.	ACU. Acupuncture needle: Huatuo brand 0.3 mm × 40 mm disposable sterile acupuncture needle. 3 weeks.	Effective rate; VAS; SFA.
Zhang 2020 (1) (52)	41/41; 45.17 ± 5.49/44.52 ± 5.27(y)	UgA. Ultrasound guidance; anesthetic: 0.5% lidocaine hydrochloride 2.5 mL; acupotomy: Hanzhang acupotomy. 3 weeks.	UgNRB. Ultrasound guidance; injection needle: No. 7; injection drugs: 2% lidocaine hydrochloride injection 2.5 mL, methylcobalamin injection 500 μg, triamcinolone hydrochloride injection 10 mg, 0.9% sodium chloride injection appropriate amount, total drug volume 5 mL. 3 weeks.	VAS; SFA.
Zhao 2023 (53)	46/46; 70.52 ± 4.52/71.45 ± 4.62(y)	UgA + ACU + traction. Ultrasound equipment: Mindray M8 portable color Doppler ultrasound system; Acupotomy: disposable; Traction training: once a day, 20–30 min/time, traction weight is 25%–33% of the patient's body weight. 4 weeks.	ACU + traction. Acupuncture: Huatuo brand acupuncture needles (0.35 mm × 50 mm; 0.25 mm × 25 mm); traction training: once a day, 20–30 min/time, traction weight is 25%–33% of the patient's body weight. 4 weeks.	Effective rate; VAS; CROM; MFV-VA/BA.
Zhong 2019 (54)	30/30; 42.8 ± 5.1/43.2 ± 4.6(y)	UgA. Ultrasound equipment: Hitachi EUB-6500 color Doppler ultrasound diagnostic instrument (5–12 MHz); Acupuncture: No. I-3 acupuncture knife. 2 weeks.	UgNRB. Ultrasound equipment: Hitachi EUB-6500 color Doppler ultrasound diagnostic instrument (5–12 MHz); injection drug: 1% lidocaine 8 mL. 2 weeks.	Effective rate; SFA; MFV-VA/BA.
Zhu 2024 (55)	45/45; 45.28 ± 4.47/45.32 ± 4.52(y)	UgA + PRF. Ultrasound guidance; acupuncture; radiofrequency therapy device: R-2000B (frequency 2 Hz, pulse width 20 ms, temperature 42°C, radiofrequency treatment 15 min). 3 weeks.	Non-UgA. No ultrasound guidance; acupuncture. 3 weeks.	Effective rate; VAS; CROM; NDI.

NA, not available; UgA, ultrasound-guided acupotomy; non-UgA, non-ultrasound-guided acupotomy; NRB, nerve root block; UgNRB, ultrasound-guided nerve root block; ACU, acupuncture; TCM, traditional Chinese medicine; PRF, ulsed radiofrequency; VAS, visual analogue scale/score; NDI, neck disability index; CROM, cervical range of motion; SFA, symptom and function assessment; MFV-VA/BA, mean flow velocity of vertebral and basilar arteries.

The interventions in the included studies involved UgA, non- UgA, and combinations with other therapies such as pulsed radiofrequency (PRF), traction, and traditional Chinese medicine (TCM). In the studies by Pu et al. (23), Cai et al. (24), Du et al. (27), and Deng et al. (25), the control groups received ultrasound-guided nerve root block (UgNRB) treatment. Additionally, in the studies by Fu and Huang (28), Zhang and Yao (51), and Huang et al. (31), the control groups received non-ultrasound-guided acupotomy or acupotomy procedures. Other studies, such as those by Wang et al. (46) and Luo et al. (42), involved comparison with combinations of non-ultrasound-guided acupotomy and other therapies, such as PRF and traction. The interventions ranged from 2 to 8 weeks, with treatment durations and frequencies varying across studies. All trials reported at least one primary outcome.

### Risk of bias results

**Figure 2:**
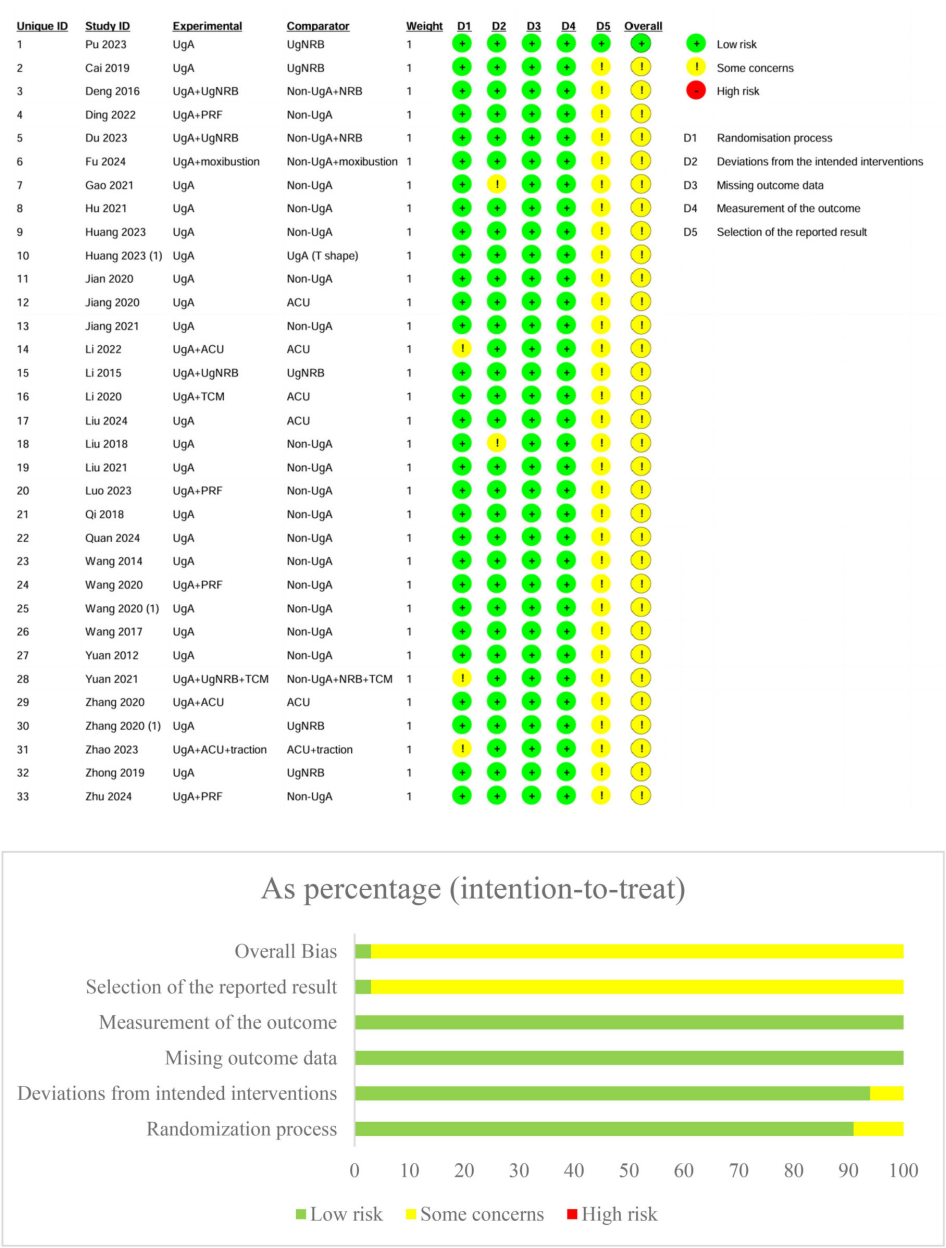
Risk of bias assessment diagram.

The risk of bias in the included studies was assessed using the Cochrane Risk of Bias 2.0 tool. In terms of the randomization process, the majority of studies were classified as having low risk [e.g., Pu et al. (23), Cai et al. (24), Deng et al. (25)], although some studies raised concerns regarding randomization, especially when allocation concealment was unclear [e.g., Jiang and Ke (35)]. The deviations from intended interventions domain showed that most studies adhered to the interventions as planned, with minimal concerns or high-risk deviations in a few cases [e.g., Du et al. (27)]. Regarding missing outcome data, a few studies had incomplete data, raising concerns [e.g., Li and Cheng (38), Yuan et al. (50)], though the majority maintained low risk. For measurement of outcomes, most studies used appropriate, blinded methods for outcome assessment, but a few studies raised concerns over the risk of detection bias [e.g., Zhang and Yao (51), Huang et al. (31)]. Selection of the reported results generally had low risk (Figure 2).

### Meta-analysis of effective rate

**Figure 3:**
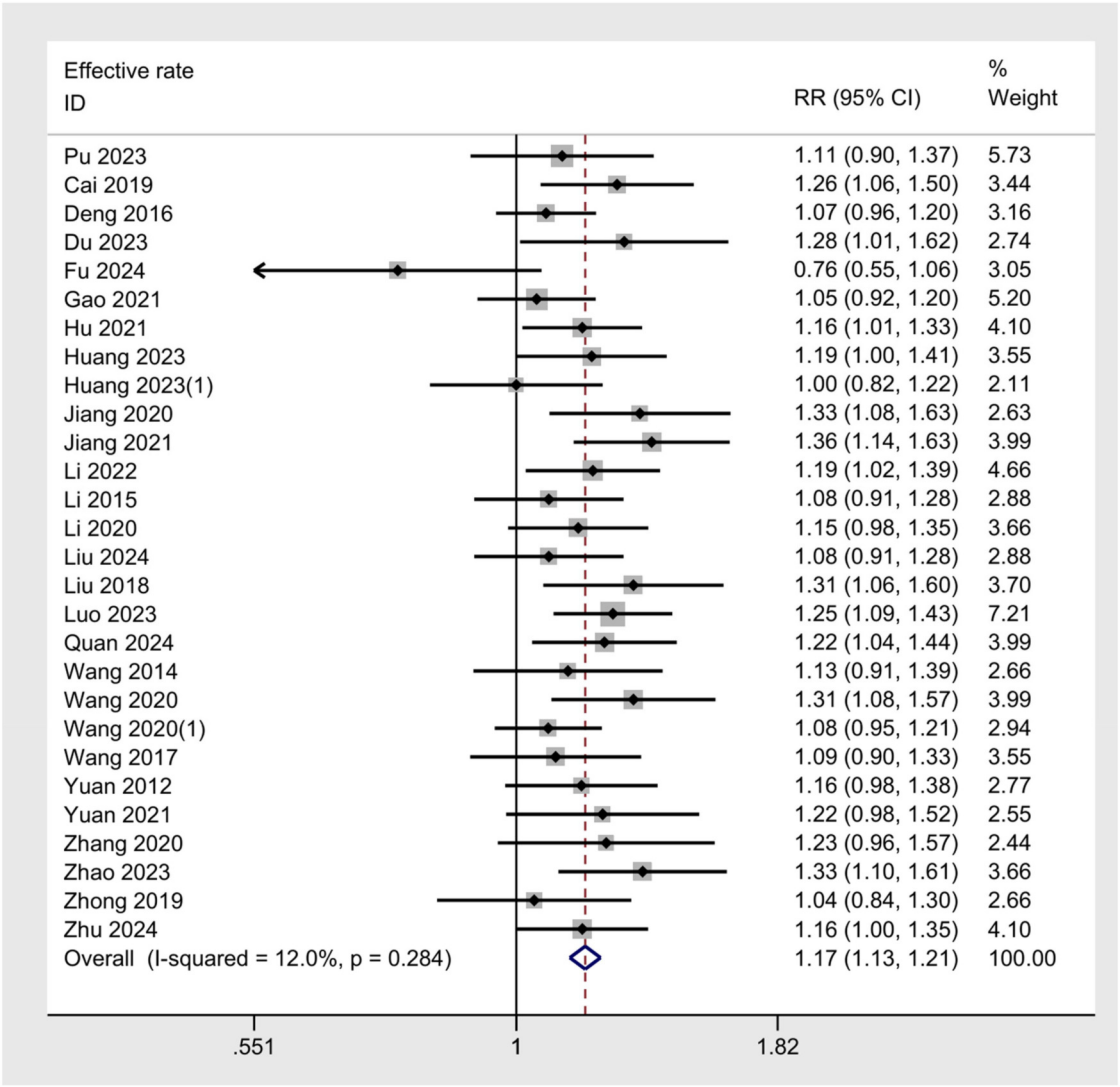
Forest plot of Effective Rate.

**Figure 4:**
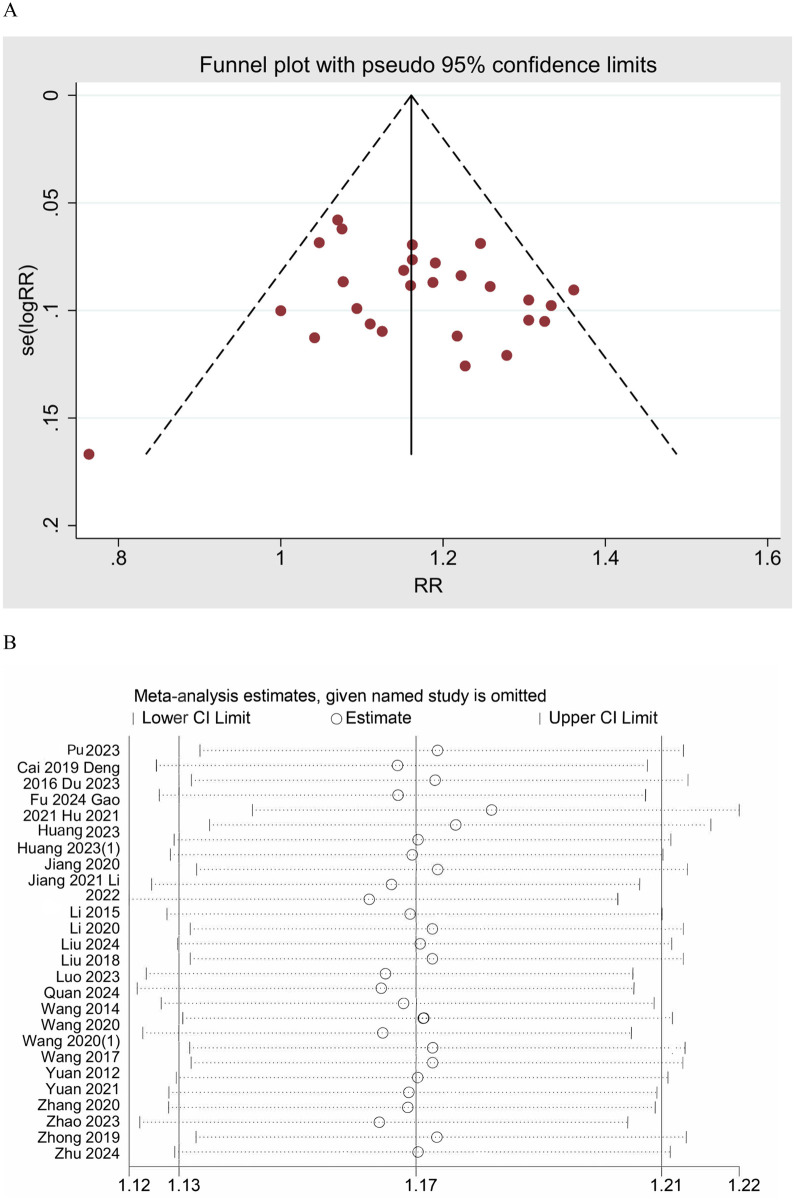
**(A)** Funnel plot of effective rate. **(B)** Sensitivity analysis of effective rate.

The meta-analysis of the effective rate across 28 studies yielded a pooled RR of 1.17 (95% CI: 1.13–1.21, *z* = 8.8, *p* < 0.001), indicating a statistically significant improvement in the effective rate for UgA compared to control treatments (Figure 3). The *I*^2^ value was 12.0%, indicating good consistency between studies. A funnel plot was generated to assess publication bias, and no clear asymmetry was observed, suggesting a low risk of bias in the published studies (Figure 4A). Additionally, sensitivity analysis was performed by sequentially omitting individual studies, and the pooled effect estimate remained stable, further supporting the robustness of the results. (Figure 4B)

### Meta-analysis of VAS scores

**Figure 5:**
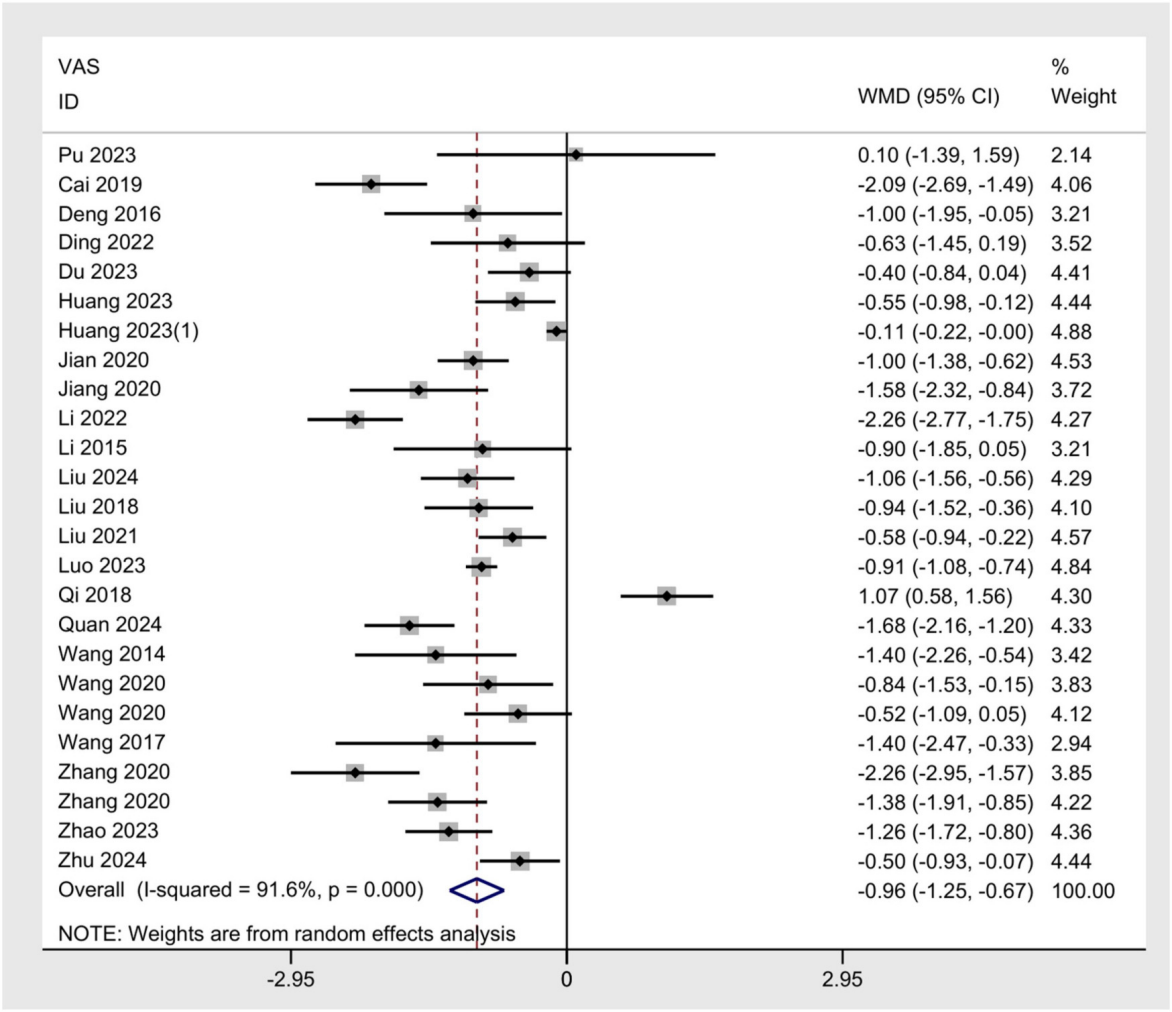
Forest plot of VAS.

**Figure 6:**
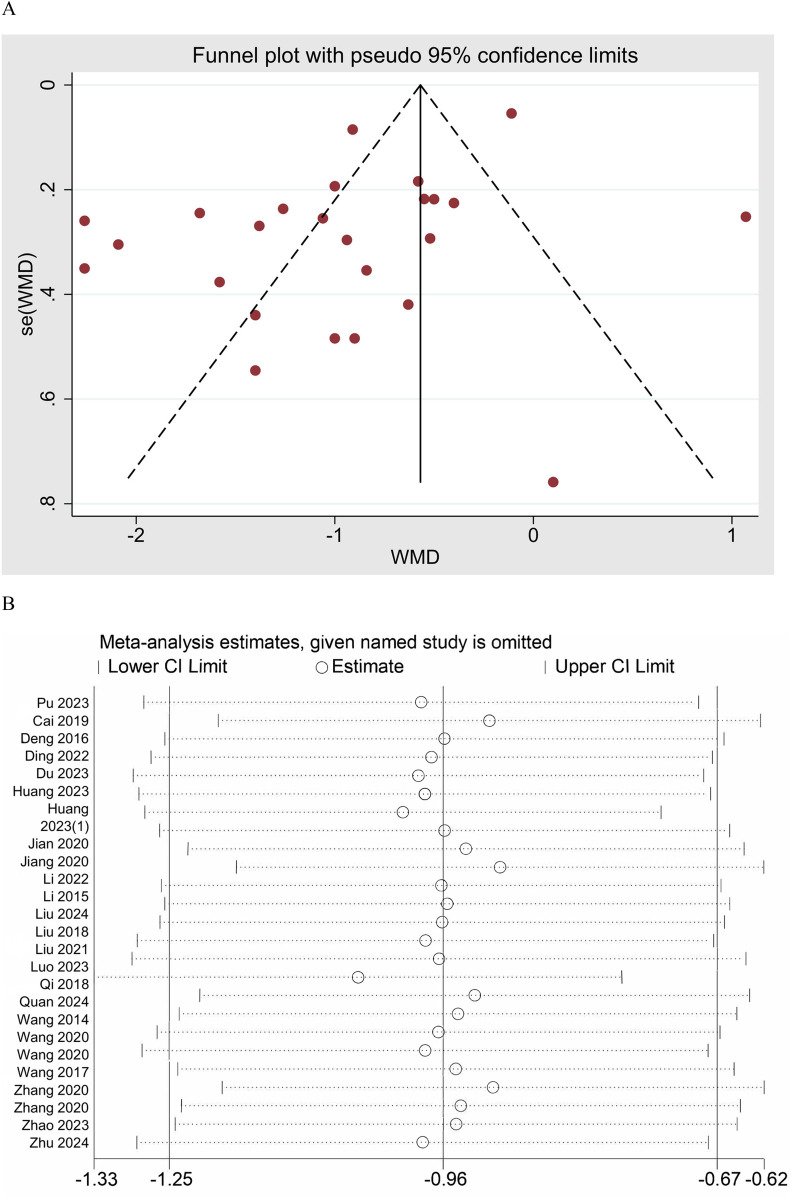
**(A)** Funnel plot of VAS. **(B)** Sensitivity analysis of VAS.

The meta-analysis of the VAS across 25 studies demonstrated a significant reduction in pain intensity following UgA compared to control interventions. The pooled WMD was −0.96 (95% CI: −1.25 to −0.67), indicating that the UgA group can significantly reduce VAS scores compared to the control group (Figure 5). Substantial heterogeneity was observed across the model, with an *I*^2^ value of 91.6%. Publication bias was assessed using both Begg's and Egger's tests. Begg's test showed no significant bias (*p* = 0.889), and Egger's test suggested the presence of some bias (*p* = 0.007), indicating potential small-study effects (Figure 6A). The trim and fill method did not impute any missing studies. The sensitivity analysis showed that excluding individual studies did not significantly alter the pooled estimate (Figure 6B).

### Meta-analysis of NDI scores

The meta-analysis of NDI scores from 10 studies showed a pooled WMD of −4.55 (95% CI: −6.72 to −2.39), indicating significant improvement in disability. High heterogeneity was observed (*I*^2^ = 94.4%). Publication bias was not significant (Begg's *p* = 0.929, Egger's *p* = 0.186). Sensitivity analysis confirmed the robustness of the results.

### Meta-analysis of CROM

The meta-analysis on CROM of 10 studies revealed a pooled SMD of 0.96 (95% CI: 0.60–1.31), indicating a significant improvement in cervical flexibility following UgA. However, substantial heterogeneity was observed, with an I-squared value of 84.5%, suggesting high variability across studies. To assess publication bias, Begg's test indicated significant bias (*p* = 0.009), while Egger's test showed no significant bias (*p* = 0.459). The trim and fill analysis imputed one missing study to address asymmetry, resulting in an adjusted random-effects estimate of SMD = 0.80 (95% CI: 0.40 to 1.21), which remained statistically significant. Sensitivity analysis revealed that the pooled estimate remained robust after omitting individual studies.

### Meta-analysis of SFA

The meta-analysis of SFA across 8 studies yielded a pooled SMD of 1.33 (95% CI: 0.85–1.81), indicating a significant improvement in symptom and functional outcomes following ultrasound-guided needling compared to controls. High heterogeneity was observed with an *I*^2^ value of 87.9%. Begg's test indicated potential publication bias (*p* = 0.013), and Egger's test suggested the presence of bias (*p* = 0.016). The trim and fill method imputed 2 potentially missing studies to adjust for publication bias. The adjusted random-effects estimate decreased from SMD = 1.33 to SMD = 1.05 but remained statistically significant, suggesting that the original effect may have been overestimated yet robust. Sensitivity analysis, excluding individual studies, confirmed the stability of the pooled estimate.

### Meta-analysis of MFV-VA/BA

The meta-analysis of MFV-VA revealed a pooled SMD of 1.13 (95% CI: 0.61, 1.64), suggesting the difference between the UgA group and the control group was statistically significant. Significant heterogeneity was observed (*I*^2^ = 87.8%). Begg's test (*p* = 0.573) and Egger's test (*p* = 0.262) did not indicate significant publication bias. Sensitivity analysis showed stable results. The meta-analysis of MFV-BA indicated a pooled SMD of 1.83 (95% CI: 0.76, 2.91), suggesting the difference was statistically significant. Notable heterogeneity was found (*I*^2^ = 95.9%). Publication bias was not detected by Begg's test (*p* = 1.000) and Egger's test (*p* = 0.928). The results remained consistent through sensitivity analysis.

### GRADE assessment

**Figure 7:**
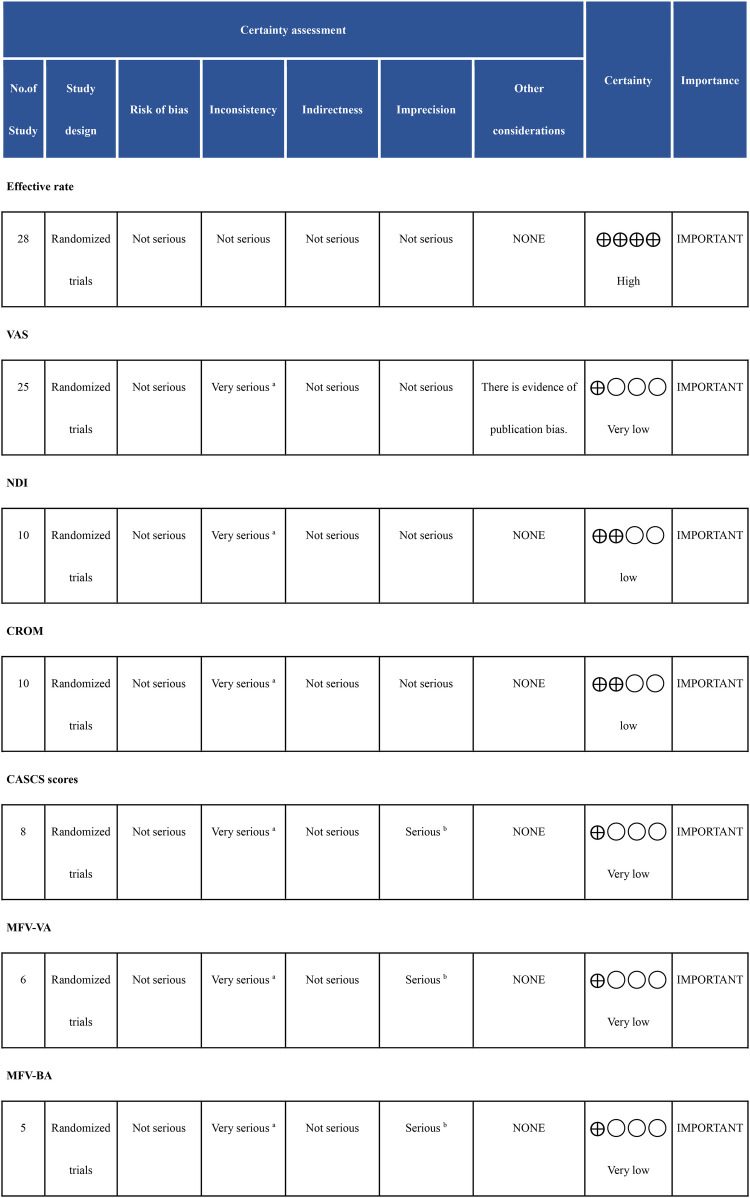
GRADE Assessment.

For the effective rate across 28 randomized trials, the certainty of evidence is high. There were no serious concerns regarding risk of bias, inconsistency, indirectness, or imprecision. In contrast, the certainty of evidence for the VAS, based on 25 randomized trials, was rated as very low. This downgrading was primarily due to serious inconsistency, as indicated by an *I*^2^ > 70%. Additionally, publication bias was identified, which further impacts the robustness of the evidence. For the NDI and CROM, both based on 10 randomized trials, the certainty of evidence is low. The primary concern was serious inconsistency (*I*^2^ > 70%), though there were no issues related to bias or imprecision. Outcomes such as SFA, MFV-VA, and MFV-BA, based on small sample sizes (8, 6, and 5 studies, respectively), also received a very low certainty rating. The reasons for downgrading included serious inconsistency and serious imprecision due to the small sample sizes and significant variability in the studies. These outcomes are still considered important but with very low certainty in their findings (Figure 7).

## Discussion

Our results demonstrates that UgA provides significant clinical benefits across multiple outcomes in cervical spondylosis. UgA significantly increased the effective rate, reduced pain intensity, and improved neck disability. Secondary outcomes confirmed notable improvements in cervical range of motion, symptoms and function, and vertebral-basilar artery hemodynamics. These findings are consistent with prior literature, confirming its positive impact (56–58). This study presents the first meta-analysis on the effects of UgA on vertebrobasilar artery blood flow velocity, seeking to elucidate potential mechanisms of this therapy. The results observed increase in vertebrobasilar artery blood flow velocity following UgA treatment. Physiologically, increases in vertebrobasilar flow could plausibly reflect improved regional perfusion and reduced ischemia of pain-generating soft tissues, thereby supporting symptom relief (59). This may be particularly beneficial for patients with cervical spondylosis who often experience vertebrobasilar insufficiency due to mechanical compression or sympathetic stimulation (60). UgA may not only address musculoskeletal dysfunction but also promote neurovascular recovery.

The consistently high heterogeneity observed in most outcomes raises important methodological considerations. This variability may reflect fundamental differences in treatment protocols, including acupoint selection, treatment frequency, and technique, as well as divergent outcome assessment methods. Furthermore, some results were detected potential publication bias, especially in patient-reported outcomes like VAS, underscores the need for cautious interpretation. These factors collectively diminish the certainty of the evidence and highlight an imperative for more rigorously designed. Implications for practice are therefore cautious. Where ultrasound equipment and trained operators are available, UgA may be considered for patients with pain and mobility limitations, with shared decision-making that prioritizes outcomes meaningful to patients (pain, function, return to activity).

Most trials assessed outcomes immediately or shortly after treatment, providing robust evidence for acute efficacy but insufficient data on long-term sustainability. Future research incorporating prolonged follow-up periods is essential to determine the persistence of benefits in pain relief, functional improvement, and hemodynamic changes. Establishing the long-term efficacy will be crucial for positioning UgA within chronic management strategies.

As most included trials were conducted in China, the generalizability of the findings to other health systems may be limited. Contextual factors such as differences in provider training, access to ultrasound equipment, usual care practices, and patient attitudes toward TCM could influence the outcomes and applicability of UgA elsewhere. Clinicians outside China should consider local feasibility and prioritize patient-centered outcomes such as pain, function, and quality of life when evaluating UgA.

## Conclusion

UgA has a higher overall efficacy compared to non-UgA and other complementary alternative therapies such as traditional acupuncture, and is beneficial for pain and disability. However, substantial heterogeneity and suspected publication bias reduce certainty. In practice, UgA may be considered for adults with CS who remain symptomatic with restricted cervical motion after conservative care, in settings with real-time ultrasound and trained operators. Future work should comprise preregistered, multicenter randomized trials—including sites outside China—with standardized UgA parameters, clearly defined comparators, blinded outcome assessment, and complete adverse events reporting.

## Data Availability

The original contributions presented in the study are included in the article/Supplementary Material, further inquiries can be directed to the corresponding author.
